# A Lethal Combination of Delirium and Overcrowding in the Emergency Department

**DOI:** 10.3390/jcm12206587

**Published:** 2023-10-18

**Authors:** Alessandra Bonfichi, Iride Francesca Ceresa, Andrea Piccioni, Christian Zanza, Yaroslava Longhitano, Zoubir Boudi, Ciro Esposito, Gabriele Savioli

**Affiliations:** 1 Department of Internal Medicine, IRCCS Fondazione Policlinico San Matteo, 27100 Pavia, Italy; alessandra.bonfichi@gmail.com; 2 Department of Emergency Medicine, Humanitas University-Research Hospital, 20089 Rozzano, Italy; irideceresa@gmail.com (I.F.C.); lon.yaro@gmail.com (Y.L.); 3 Department of Emergency, Fondazione Policlinico Universitario A. Gemelli, IRCCS Fondazione Policlinico San Matteo, 00168 Roma, Italy; andrea.piccioni@policlinicogemelli.it; 4 Italian Society of Pre-Hospital Emergency Medicine (SIS-118), 74121 Taranto, Italy; christian.zanza@live.it; 5 Department of Anesthesiology and Perioperative Medicine, University of Pittsburgh Medical Center, Pittsburgh, PA 15260, USA; 6 Department of Emergency Medicine, Dr Sulaiman Alhabib Hospital, Dubai 2542, United Arab Emirates; zoubir.boudi@gmail.com; 7 Nephrology and Dialysis Unit, ICS Maugeri, University of Pavia, 27100 Pavia, Italy; ciro.esposito@unipv.it; 8 Emergency Medicine and Surgery, IRCCS Fondazione Policlinico San Matteo, 27100 Pavia, Italy

**Keywords:** delirium, emergency department, dementia, acute confusion, elder, epidemiology, etiology, assessment, diagnosis, emergency medicine, emergency care, risk factors, overcrowding

## Abstract

Delirium is a common public health concern that significantly impacts older patients admitted to the Emergency Department (ED). This condition is linked to adverse outcomes such as reduced long-term functionality, higher mortality rates, extended hospital stays, and increased medical costs. The identification of risk factors is crucial for the early recognition and management of delirium in ED patients. Aging, cognitive decline, polypharmacy, and sensory impairment are some of the most common general risk factors described in the literature. Although validated delirium assessment tools already exist, they are not practical for the fast-paced ED environment because of their extended evaluation period or specialized training request. Moreover, clear guidance is needed to select the most suitable tool for detecting delirium, balancing between the accuracy and the swiftness required in an overcrowded, high-stress, and understaffed healthcare setting. This narrative review aims to analyze the updated literature on delirium risk factors in older ED patients and focuses on the methods for better screening, managing, and treating this condition in the ED.

## 1. Introduction

Delirium is an underrecognized public health condition that commonly affects older patients who are admitted to the Emergency Departments (ED) [1]. Delirium is associated with multiple poor outcomes, including decreased long-term functioning, increased mortality [2], and length of hospital stay, as well as medical costs [3,4,5,6]. Managing delirious patients in the ED is challenging. They may be prone to fall, remove intravenous catheters or endotracheal tubes, exhibit intolerance to invasive procedures, or become aggressive, thus endangering themselves and those providing care [7,8]. Despite the high morbidity and mortality associated with delirium, emergency healthcare providers underrecognized it [9,10,11]. The difficulty in recognizing delirium begins at triage, even with advanced systems that best adapt to the increasing complexity of older patients and the rise in crowding [12,13]. The difficulty persists throughout the entire patient’s process. According to previous studies, ED physicians diagnosed delirium in only one out of six delirious patients [14]. ED clinicians and nurses must be aware of how to optimize delirium recognition, prevention, and management. Most of the studies evaluating delirium risk factors have been conducted in other medical contexts, such as the intensive care unit (ICU) or hospital wards, but they have limitations in the ED. Firstly, previous researchers have often recruited patients 48 h after admission. Given its fluctuating nature, the delirium in a patient after 48 h of hospitalization could be completely different from the status of delirium of the patient’s arrival in the ED [15]. Secondly, the urgency of the ED environment and the heterogeneity of the ED population make it challenging to establish the appropriate delirium assessments. Validated tools for assessing delirium in various settings have been developed, but most of them require specialized training or longer evaluation times that may not be practical in the ED [16]. By reviewing the updated literature, our aim is to analyze the risk factors associated with the occurrence of delirium in older ED patients and methods developed to screen, manage, and treat this condition.

## 2. Materials and Methods

For this narrative review, we conducted a comprehensive search spanning from January 1981 to April 2023. Study selection was made encompassing major scientific databases, namely, Pubmed, Scopus, Medline, Embase, WebScience, and Google Scholar. Utilizing the MeSH database, our initial search yielded a total of 1876 articles that matched the keywords delirium; emergency department; dementia; acute confusion; elder; epidemiology; etiology; assessment; diagnosis; emergency medicine; emergency care; risk factors; overcrowding Subsequently, a second screening narrowed down the selection to 550 relevant papers from which we excluded meeting abstracts, books, manuscripts unavailable, original papers without abstracts, and brief reports and kept only relevant articles related to oncology and emergency medicine, critical or intensive care medicine, and acute medicine. Moreover, reference lists in each identified article were reviewed to find any relevant additional publications. In the end, a total of 116 papers were analyzed in this clinical review.

## 3. Results

### 3.1. Definition

Delirium is an acute disruption in the brain networks defined by the Diagnostic and Statistical Manual of Mental Disorders, fifth edition (DSM-5), resulting in fluctuating changes in the level of consciousness, attention, and cognition [3]. The disorder should appear within a short period of time (hours or days) [5] and represents a change from the patient’s baseline condition. Five core domains have been described: cognitive deficits; attentional deficits; circadian rhythm dysregulation; emotional dysregulation; and psychomotor dysregulation, which confer the various phenotypic presentations [17]. In order to diagnose delirium, these disturbances should not be attributable to other neurocognitive disorders. Clear evidence indicating that they result from a medical condition, exposure to toxins, or a combination of multiple causes is required [18]. According to clinical features, three psychomotor subtypes have been described: hyperactive; hypoactive; and mixed subtypes. Hyperactive, delirious patients present increased psychomotor activity. They appear restless, anxious, agitated, and aggressive. Hypoactive delirium is characterized by somnolence or decreased mental status. Patients with mixed subtypes exhibit fluctuating levels of psychomotor activity (hypoactive and hyperactive). According to Han et al. [19], hypoactive and mixed-type delirium appear to be the predominant subtypes in older patients. Each psychomotor subtype is hypothesized to have a different underlying pathophysiological mechanism and underlying etiology. For example, delirium caused by alcohol withdrawal is more likely included in the hyperactive subtype, whereas delirium caused by a metabolic derangement is more likely to be the hypoactive subtype [20]. A classification of delirium based on the etiology must be mentioned. As an example, postoperative delirium (POD) is a specific subtype that occurs after surgical procedures. It shares the characteristics of delirium, but it is associated with surgery-related factors. It often presents in the hypoactive form.

While not meeting the full criteria for diagnosis of delirium, Subsyndromal Delirium (SSD) represents a milder form of cognitive impairment, especially in the elderly. Patients affected by SSD may exhibit delirium-like features, such as changes in cognition and attention, and less severe psychotic and motor symptoms.

Because of this heterogeneity, recognizing delirium in the ED can be challenging because of the subtle changes in patient behavior that can be easily overlooked in an overcrowded environment. In addition to clinical difficulties, there are challenges associated with crowding, such as a shortage of doctors and nurses available to meet healthcare needs [21,22,23,24].

### 3.2. Delirium and Dementia

Delirium and dementia are the most common causes of cognitive impairment among older adults, but they represent two different clinical entities [18]. The presence of delirium has been demonstrated to be a strong risk factor for incident dementia and cognitive decline in patients who are 85 years and older [25]. Delirium is characterized by a rapid onset from hours to days. Fluctuations and reversibility are key features. On the other side, dementia is characterized by a gradual, irreversible decline in cognition occurring over months or years. Unlike delirium, altered levels of consciousness, inattention, perceptual disturbances, and disorganized thinking are not commonly observed in patients with dementia. Nevertheless, the clinical features of delirium and dementia may overlap, making diagnosis challenging [18]. It is reported that almost 20–30% of older hospitalized patients present dementia, often undiagnosed, frequently with superimposed delirium [26]. Clinical situations, such as severe or terminal dementia or dementia with Lewy bodies (DLB), may exhibit overlapping symptoms of both delirium and dementia. Similarities for the former include disorganized thinking and altered levels of consciousness, while the latter can show rapid deterioration and fluctuations in cognition, along with inattention [1,27,28]. Due to the difficulty in distinguishing these two conditions, the knowledge of the baseline mental status may be crucial to diagnosing delirium in patients with dementia [29]. In the ED, the differential diagnosis might be challenging due to the lack of obtaining accurate medical information about the patient’s baseline cognitive function, especially when caregivers are not available. This happens in up to 40% of the elderly, especially when patients arrive by ambulance and/or from long-term care facilities. The patient’s delirious state, together with the presence of psychiatric illness or dementia and the layout of the ED, makes it difficult to gather verbal reliable medical history [30,31].

### 3.3. Epidemiology

Delirium is a common condition among hospitalized elderly patients. The majority of studies focus on a limited number of participants and have been generally estimated among specific patient populations and settings [32]. The wide range of prevalence values reflects the interaction of different patient types and the quantity and severity of the precipitating risk factors [5]. Furthermore, a significant number of studies excluded patients with pre-existing cognitive impairment or dementia, which suggests that the reported incidence rates are likely underestimated. The prevalence of delirium varies across different settings, with ICUs often experiencing the highest rates [5,6,33]. According to acute hospital settings, previous studies demonstrated that approximately 20% of inpatients experience delirium [32,33,34]. Relating to the surgical setting, the incidence of delirium after surgery varies depending on the complexity of the procedure and the patient’s health status. In patients who underwent minor elective surgery, the occurrence of delirium is typically low. In patients who underwent major surgery, the incidence can exceed 20%, particularly in emergency situations [5]. Delirium is also a significant concern in palliative care, with a prevalence estimated at around 4–12% in the community and 6–74% in palliative care units [5].

Most of the epidemiology studies of delirium in the ED present limitations due to the unique nature of this healthcare setting and its high turnover. Many studies enrolled patients within 24–48 h of hospitalization. Since delirium can resolve in 24 h or less, patients classified as nondelirious in the hospital setting may have been delirious in the ED [35].

The prevalence of delirium in ED is considered significant among adults over 65 years. Emerging research indicates that delirium occurs in 7–20% of cases in ED, and the condition goes undiagnosed in approximately 57–83% of these instances [36]. Delirium incidence increases with the patient’s age, disease severity, and associated complications [37]. The incidence rate of delirium in ED ranges from 7 to 10% of patients aged >/= 65 years [14]. Boucher et al. [36] showed that the recognition of incident delirium in the ED has not improved over time. Their study conducted in five ED departments showed that 84.6% of patients with a positive assessment tool for delirium were unrecognized and discharged at home without appropriate care [38].

### 3.4. Outcomes in ED

The presence of delirium in the ED has been linked to increased mortality rates at 6 months, as well as accelerated functional decline at 18 months [39]. Kakuma et al. showed that the mortality rate was higher among ED patients whose delirium went unnoticed by the attending physician, in comparison to ED patients whose delirium was identified and patients without delirium. According to their study, delirium remained a statistically significant predictor of 6-month mortality even when adjusted for age, sex, functional level, and number of medications [38]. Delirium is associated with prolonged hospital stay, cognitive decline, increased risk of falls, and overall dependency that can require institutional care [40,41,42]. Furthermore, delirium has a considerable economic impact on the healthcare systems [43].

### 3.5. Aetiology and Pathogenesis

Delirium is a multifactorial syndrome that involves a complex interrelationship between baseline patient vulnerability (e.g., cognitive impairment or other comorbidities) and a superimposed acute stressor [44,45]. Wilson et al. explained the mechanism behind the pathogenesis of delirium [5]. Firstly, the brain network is impaired by aging and neurodegeneration because of decreased physiological reserve and increased susceptibility to disruption in electrolyte imbalances, glucose, or oxygen levels. Hormonal changes, inflammation, and abnormalities in neurotransmitter function (e.g., acetylcholine, dopamine) also contribute to this phenomenon. Furthermore, the aging process alters brain vascularization, leading to impaired perfusion and disruption of the transport of important plasma proteins as well as a leakiness of the blood–brain barrier (BBB). Compromised BBB integrity, reduced cerebral blood flow, and impaired clearance of toxic substances are all contributing factors to the development and progression of neurodegeneration [46]. Additionally, compromised BBB function increases the permeability of systemically administered medications with a deleterious impact on the brain [5]. Animal model research demonstrates that neurodegeneration primes both microglia and astrocytes, leading to an enhanced production of pro-inflammatory responses [47]. In response to various triggers such as infection, injury, or underlying medical conditions, immune system inflammatory mediators, including cytokines and chemokines, are released [45]. These molecules can disrupt the BBB, being responsible for synaptic dysfunction and cognitive symptoms of delirium [48].

Understanding the causes of delirium is crucial for its prevention and appropriate treatment. Several factors have been linked to the onset of delirium, such as sepsis, trauma, surgery, medication changes, hypoglycemia, dehydratation, and organ failure.

Sepsis is a worldwide leading cause of morbidity and mortality, and the central nervous system is particularly susceptible to dysfunction [45,49]. Infections are the most common cause of delirium identified in ED studies and are responsible for 30 to 40% of cases. A prospective study conducted in four EDs showed that infection (pneumonia and urinary tract disease) and neurological diseases (ischemic stroke, intracranial hemorrhage, or intracranial mass) were the most common etiologies, followed by metabolic–electrolytic (renal/uremic pathologies and blood glucose disorders), cardiopulmonary, and gastrointestinal diseases [50].

Given the brain’s high energy demands, metabolic alterations (e.g., hypoxia, dehydration/electrolyte imbalance, or hypoglycemia) can precipitate delirium [51]. Low regional cerebral oxygenation in critical brain areas, such as the caudate nucleus or frontal cholinergic pathways, can cause attentional deficits and delirium [52]. The basal ganglia may be especially susceptible to these effects because their blood supply comes from small perforating vessels [45]. Furthermore, hypoxia-induced inflammation and oxidative stress can further contribute to the pathophysiological processes underlying delirium. Among other causes, dehydration (e.g., decreased thirst mechanism, overuse of diuretics, or swallowing difficulties) is a common precipitating factor for delirium, leading to cerebral hypoperfusion [7].

Urinary retention must be considered. Delirium may be influenced by bladder distension, as the tension on the bladder wall can lead to heightened sympathetic tone and an increase in catecholamine release [7].

Iatrogenic factors (e.g., bladder catheters, physical restraints, or drugs) are the most common reversible cause of delirium [53]. Drug toxicity contributes to approximately 12–39% of all cases of delirium. In addition, polypharmacy has an additive and synergistic role with drugs in causing delirium in the elderly [44,53]. Delirium can be particularly triggered or worsened by drugs with anticholinergic properties as well as by sedative–hypnotics (benzodiazepines) [1,5,54]. The mechanism of action of anticholinergic drugs enables their therapeutic use in various clinical conditions, including Parkinson’s disease and urinary incontinence. The cholinergic system is involved in arousal, attention, memory, and rapid eye movement (REM) sleep. Evidence from experimental studies and clinical observations suggested that anticholinergic drugs can cause physical and mental impairment in elderly people [55]. The hypothesis that a deficiency in cholinergic function may play a role in the development of delirium stems from the association between the neurotransmitter acetylcholine and cognitive processes affected during delirium, including attention, sleep, and memory [56,57]. Benzodiazepines can accumulate in lipid tissue, with a prolonged half-life in the elderly. Any rapid reduction in benzodiazepine dose can produce a pro-excitatory state. Due to their extended action and the heightened sensitivity of older people, benzodiazepines may be responsible for the onset of delirium [53].

### 3.6. Risk Factors

Defining delirium risk factors would allow clinicians to identify high-risk patients and target them for focused delirium prevention [58]. As underlined above, the pathophysiology of delirium revolves around the interplay between an existing predisposition and the impact of an acute stressor. Therefore, there are two categories of factors involved: predisposing risk factors; and precipitant risk factors.

The first group includes conditions that exist before the onset of delirium. They are related to the person’s baseline health status, cognitive function, or, especially in geriatric patients, physiological vulnerabilities due to frailty [59]. Several studies have identified advanced age as a significant independent predisposing factor for delirium in hospitalized patients. As reported by Maldonado et al., the risk of delirium increases with age [17]. As well as the aging process, cognitive impairment showed a strong linear association with delirium risk [5,60,61,62]. According to Elie et al., psychiatric illness, such as depression, has been believed to be related to risk factors, but results appeared to be controversial [61]. Polypharmacy, sensory impairment, and social isolation have been identified as predisposing factors that increase vulnerability among frail geriatric patients [59]. Other vulnerability factors reported in the literature include the presence of comorbidities, history of alcohol abuse [63], malnutrition, and opioid or benzodiazepine use at home [35,54].

Precipitating risk factors include acute events that trigger the onset of delirium in individuals who are predisposed. These factors are often temporary and are reversible, and they may directly or indirectly affect brain function [5]. They might involve acute illness, medication changes, surgery, infections, environmental changes [59], mechanical ventilation [64] and uncontrolled pain [6].

Most of the studies concerning delirium risk factors have been conducted in the ICU, surgery, or other inpatient settings, and they cannot be generalized to ED patients. A systematic review performed by Zaal et al., including 33 studies, identified risk factors for delirium in critically ill adults in the ICU. Strong evidence indicates that age, dementia, systemic arterial hypertension, pre-ICU emergency surgery or trauma, Acute Physiology and Chronic Health Evaluation (APACHE) II score, mechanical ventilation, and metabolic acidosis contribute to the risk of developing delirium [65]. Furthermore, oversedation (especially with the use of benzodiazepines) commonly occurs in the ICU and is associated with worse clinical outcomes, such as longer time on mechanical ventilation, prolonged stay in the ICU, and an increased risk of developing delirium [66].

Systematic reviews and other meta-analyses [67] identified respectively risk factors and outcomes for delirium overall in the ICU. By reviewing the relevant literature, Krewulak et al. [68] highlight evidence suggesting that delirium subtypes may exhibit differences in their associated risk factors and outcomes in an adult ICU population. According to their research, patients with hypoactive delirium have a higher mean age and mortality compared to other subtypes of delirium.

According to surgical settings, systematic review and meta-analysis evaluated the risk factors for delirium in vascular surgical populations [69], including smoking, longer duration of surgery, American Society of Anesthesiologists (ASA) physical status score > 2, and presence of postoperative complications as other risk factors associated with delirium. POD has been described as a common and underestimated complication following surgery. Risk factors associated with POD include the presence of comorbidities (congestive heart failure, hypertension, kidney disease, anemia, diabetes, and pulmonary disease), pre-existing dementia, procedure-related risk factors (emergency surgery or duration of certain surgical procedures), anesthesia-related factors [70], and the use of perioperative medications, such as opioids or benzodiazepines and preoperative infections. Furthermore, patients who received less effective pain treatment after hip fracture surgery were more likely to experience POD [51].

Ahmed et al. [71] focused on older inpatients admitted to acute hospital units. According to their study, factors significantly associated with delirium in this setting include infection, ‘high-risk’ medication use, diminished activities of daily living, immobility, sensory impairment (poor vision), urinary catheterization, urea and electrolyte imbalance, and low albumin as well as known factors such as dementia, older age, and co-morbid illness.

### 3.7. Risk Factors in the ED

None of the previous studies evaluated delirium risk factors in geriatric patients in the ED. As well described by a recent systematic review, no differences between an incident and prevalent delirium were considered in previous studies [15]. As defined, incident delirium refers to patients who were initially nondelirious but subsequently developed it either during their stay in the ED or during hospitalization. Prevalent delirium refers to patients who exhibit delirium at the time of assessment or evaluation without considering whether they were initially delirious or not. Since previous systematic reviews have not considered a well-defined differentiation between prevalent and incident delirium, the interpretation of their findings may be limited.

Similar to findings in other hospital settings, general risk factors, such as advanced age, pre-existing dementia, alcohol or drug withdrawal, hearing impairment, acute illness, comorbidities, poor sleep, and sedation, were found to be associated with delirium development in the ED [19].

On the other hand, some features are typical of the ED setting. Environmental factors must be considered. The nature of the ED environment amplifies the risk for delirium development because of several factors, such as the stressful and unfamiliar environment of the ED, sleep disturbances, sensory deprivation, and lack of personalized care [7,72,73]. Other factors include the absence of orientation items (e.g., legible clocks, reading glasses, hearing aids), inadequate access to natural light due to the lack of windows, and elevated noise levels. Furthermore, the limited interaction with familiar people may disorientate old patients.

Regarding the ED environment, overcrowding may be taken into consideration. Overcrowding is the result of several internal and external factors, including reduced access to hospital beds and shortages of hospital staff. It reflects the mismatch of supply and demand in the healthcare system [74,75,76]. Due to the Coronavirus pandemic, this phenomenon has increased during the last few years, becoming a growing global problem [77]. Overcrowding has been associated with adverse patient outcomes, including higher mortality rates [78] and serious consequences on seniors, including the onset of delirium [36]. In a busy healthcare department, limited time for effective patient communication can result in incomplete information exchange and subsequent issues, like misunderstandings, heightened anxiety, and confusion, especially in elderly patients. Prolonged ED length of stay (ED LOS) is another consequence of crowding excess. ED LOS is defined as the time elapsed between the initial triage registration and physical departure from the ED [79]. There is a significant association between incident delirium and ED LOS, as well as between delirium and time spent in the ED hallway [73,80,81,82]. An Italian article explored the relationship between ED LOS and the occurrence of delirium in older medical patients [81]. The authors showed that patients who spent more time in the ED (length of stay longer than 10 h) were observed to have an increased risk of experiencing delirium during their hospitalization. This implies that avoiding ED boarding time may be crucial for delirium prevention strategy [31].

### 3.8. Prevention

Prevention program strategies have demonstrated effectiveness in reducing both the incidence of delirium and the subsequent risk of adverse outcomes [18,41]. Strategies such as nonpharmacological interventions (e.g., optimizing sleep, promoting mobility, providing orientation cues) and careful medication management can help reduce the risk of developing delirium. Healthcare professionals should be trained in recognizing the signs and symptoms of delirium to facilitate prompt diagnosis and appropriate management [83]. These interventions emphasize decreased use of psychoactive medications such as anticholinergic drugs (including tricyclic antidepressants and typical antipsychotics), sedative–hypnotics (benzodiazepine, zolpidem), corticosteroids, and polypharmacy (more than four medications). Ensuring the patients’ daily functioning by supplying hydratation, mobility assistance, hearing access devices, and avoiding bladder catheters if not necessary could be helpful [1,84]. Visual measures (e.g., the presence of clocks in the room) can help patients’ self-orientation. According to the National Institute for Health and Care Excellence (NICE) guideline, avoiding moving people between wards or rooms unless necessary and promoting a normal sleep–wake cycle can help to reduce the risk of developing delirium. Mobility should be undertaken with precautionary measures against injury, like the use of low beds and physiotherapy. Ensuring adequate fluid intake, optimizing oxygen saturation, and starting appropriate pain management in any patient in whom pain is identified or suspected (especially older people with difficulties in communication because of dementia) are other important preventive measures [84,85]. According to the Cochrane database, there is moderate evidence regarding the benefit of nonpharmacological interventions to prevent delirium in hospitalized adults. They are estimated to reduce the incidence of delirium by 43%, with no evidence of an effect on mortality [41].

Sangil Lee et al. conducted a systematic review of prevention studies [86]. Their aim was to evaluate interventions that can prevent incident delirium or shorten the prevalence of delirium duration in the ED. According to their research, few interventions were found to reduce incident delirium in ED. Among these, the use of melatonin in overnight stay could be effective in reducing delirium incidence, but it did not decrease delirium severity, length of hospital stay, and mortality [87]. Secondly, the placement of the Foley catheter and the use of high-risk medications in the ED correlated with an increase in the duration of delirium [88]. However, while delirium has been increasingly recognized as a preventable source of morbidity and mortality, interventions are implemented infrequently, and little attention in terms of resource allocation for clinical care is given [43]. The Society for Academic Emergency Medicine Geriatric Task Force identified cognitive assessment as an area of ED with considerable quality care gaps. For this reason, they recommended the use of a screening cognitive assessment that, if found to be abnormal, should be followed by further evaluation to assess the presence of delirium [89].

### 3.9. Assessment and Diagnosis of Delirium in ED

Diagnosis is important to identify precipitating causes and implement management and treatment of delirium [90]. The diagnosis is often not formally made in ED [91]. A working group of experts developed an open-access web-based tool available on the American College of Emergency Physicians (ACEP) device app to assist ED physicians in the care of these patients. The ADEPT tool (Assess, Diagnose, Evaluate, Prevent, and Treat tool, ACEP, Irving, TX, USA) is the newest resource designed to provide quick access to a basic clinical guide, is easily accessible and can be used during patient care.

Life-threatening causes should be considered, such as hypoglycemia, hypoxia, hyperthermia, intracerebral hemorrhage, meningitis, encephalitis, epilepsy, myocardial ischemic attack, Wernicke disease, electrolyte imbalance, and drug toxicity.

Once life-threatening causes have been ruled out, the next step is to establish if there has been a change from baseline mental status. The process involves a bedside clinical assessment of the patient. Key diagnosis features include an acute change from baseline awareness and a fluctuating course. Supportive features include the level of attention, arousal, the presence of other cognitive deficits (e.g., memory impairment, disorientation), hallucinations, psychomotor disturbance, and emotional lability [83]. Collecting information on patients’ history and medication is crucial, including any over-the-counter drugs, alcohol consumption, illegal substance abuse, recent changes or non-compliance with medication, and missed doses. Particular attention must be paid to high-risk medications such as sedatives, steroids, antihistamines, anticholinergics, tricyclic antidepressants, muscle relaxants, and opioids.

Nevertheless, in the ED settings, physicians may not have adequate time to perform in-depth delirium assessments due to the chaotic environment and the lack of a validated and brief instrument for delirium identification [30,39]. To identify delirium among older adults in ED, a variety of tools have been proposed [85,92,93]. It is important to specify that the ideal time duration to apply a delirium scale in an overcrowded ED can vary depending on several factors (e.g., patient collaboration). The choice of a delirium scale should balance the need for accuracy with the limited time and resources available in a busy ED. The most widely used tool is the Confusion Assessment Method (CAM), validated in a higher number of studies [83,94]. The CAM comprises four components: (1) sudden onset of changes in mental status and fluctuating course; (2) inattention; (3) disorganized thinking; and (4) a modified level of consciousness [1]. Patients who meet the criteria for delirium present both feature 1 and 2 and either feature 3 or 4. The CAM sensitivity and specificity are close to 86% and 93%, respectively. Since CAM can take 5 to 10 min to be performed, it is not feasible for the ED [40]. The CAM-ICU takes less than 3 min and is an adaptation of the CAM developed for ICUs specifically. It can be used to assess delirium features in mechanically ventilated or non-verbal patients [95]. CAM ICU uses the same features of CAM with the difference that feature 1 includes acute mental changes or fluctuating courses. A patient must present features 1 and 2 and either feature 3 or 4 to meet delirium criteria [1]. In order to quickly recognize delirium and prevent its complications, other brief screening tools have been created to face emergency setting necessities. Among them, the Brief Confusion Assessment Method (bCAM) is a modified CAM designed for noncritically ill patients, and it takes 1–2 min. Similar to CAM, it includes four features: acute onset and fluctuating course; inattention; altered level of consciousness; and disorganized thinking. According to Han et al. [96], bCAM was highly specific, and patients with a positive bCAM were 20 to 25 times more likely to experience delirium compared to those with a negative bCAM. Nevertheless, this tool is validated for Critical Units. Larger, multicenter trials should be needed to confirm these findings and to determine the effect of these assessments on delirium recognition in the ED.

Since family members are critical to knowing a patient’s baseline status, which is the key to detecting acute changes in cognitive function [30], the family CAM (FAM-CAM) [97] has been created. It is a version of the CAM developed for family members and caregivers that consists of 11 items, including changes in attention, speech, arousal, and orientation, and can help ED providers in the recognition of delirium.

According to the Scottish Intercollegiate Guidelines Network (SIGN) [93], the 4AT shall be used to identify patients with probable delirium in both ED and acute hospital settings. It is a tool based on four items: Alertness, abbreviated mental test-4 (age, DOB, place, year), attention (months of the year backwards), acute change or fluctuating course. Gagnè et al. evaluated the 4AT in four Canadian EDs as a screening method to recognize cognitive impairment in functionally semi-independent and independent >/= 65 years old patients spending a minimum of 8 h in the ED. Due to its quick administration time (less than 2 min) and no training requirement, 4AT allows for systematic screening of patients at risk of delirium without significantly increasing the workload of the ED staff [26,98].

Delirium Triage Scale (DTS) is another brief tool created to quickly rule out delirium (<20 s). It is the first step of a two-step delirium monitoring process for busy clinical environments. If negative, no additional testing is needed, but in the case, it is positive, more specific assessments are needed to rule out delirium.

Among other tools, the Delirium Rating Scale (DRS) and its revision DRS-R-98 must be mentioned. They were designed to diagnose and evaluate the severity of delirium symptoms, and they have been approved for a variety of patient populations, including surgery or critical care. One challenge associated with these scales is their complexity, as they necessitate the training of skilled professionals. For this reason and its extended submission time (10 min), its application can be difficult in the emergency environment [83].

As identified by the researchers, short screening tools for delirium validated in the ED are crucial to help ED providers identify older attendees with cognitive vulnerability and trigger appropriate care pathways, as well as further education of emergency professionals about delirium recognition [16,26]. Further research may prove valuable in determining the optimal balance between a rapid diagnostic tool and the effective diagnosis of delirium.

### 3.10. Management of Delirium

Once delirium is detected, further evaluation is needed to prevent the worsening of symptoms and to maintain patient safety [83]. Efforts should focus on protecting airways and preventing aspiration, maintaining hydratation and nutrition. Evidence supporting the significant benefits and outcome improvement from pharmacological treatment is limited [40]. Nevertheless, if nonpharmacologic management is unsuccessful, pharmacologic treatment may be necessary to preserve patient safety while avoiding oversedation. It is important to underline that even low doses of antipsychotics and benzodiazepines could increase adverse effects such as prolonged sedation or paradoxical agitation in elderly people. Antipsychotics and benzodiazepines are the most frequently utilized drugs to manage acute agitation in ED settings. Agent combination is common [84,99]. Nevertheless, antipsychotic and sedative medications have been found to prolong the duration of delirium and worsen clinical outcomes [83]. Furthermore, certain antipsychotic drugs are not approved for dementia-related psychosis because of increased mortality risk in elderly people.

Antipsychotics are classified into first-generation or typical agents and second-generation or atypical agents [100]. All first-generation antipsychotics present quinidine-like cardiac effects resulting in prolonged QT, with potential dysrhythmias [101]. Haloperidol exerts pharmacologic effects through D2 receptor and alpha 1 adrenergic receptor antagonism, and it has little activity on histamine-1 or muscarinic receptors. Single doses of haloperidol between 5 and 10 mg are generally well tolerated. Haloperidol should be avoided in favor of atypical antipsychotics in patients with Parkinsonism. Droperidol is an analog of haloperidol developed in 1961, with a shorter half-life. Its onset of action ranges from 15 to 30 min. Droperidol is an effective and safe medication in the treatment of nausea and headache and is approved by the FDA as a postoperative and postprocedural antiemetic [102].

Because of longer clinical experience, haloperidol is the standard therapy, but the new atypical antipsychotic has shown fewer side effects, such as extrapyramidal symptoms, compared to the first-generation agents. There is limited experience with second-generation antipsychotic use in the ED, but a growing number of studies suggest their effectiveness. Olanzapine is a second-generation antipsychotic that exerts prominent antagonism on muscarinic receptors. It has been approved by the FDA for the management of acute agitation in schizophrenia. Despite not having formal approval for this use, olanzapine is employed in EDs to control nonspecific agitation [100]. Similar to olanzapine, ziprasidone was approved by the FDA for the treatment of schizophrenia and bipolar patients, and it has been evaluated for the treatment of nonspecific agitation in Eds. Ziprasidone is a potent D2 antagonist, and it exerts a significant antagonism effect on alpha-1 and H1 receptors, with limited activity on muscarinic receptors. It has a slower onset of action but similar efficacy in controlling agitation compared to other antipsychotic agents. One randomized trial conducted in the ICU compared haloperidol and ziprasidone with placebo [103]. The primary endpoint was the number of days alive without delirium or coma during the 14-day intervention. The use of haloperidol or ziprasidone compared with a placebo had no significant effect on the primary endpoint [104]. Among second-generation antipsychotics, data supporting the use of risperidone in acute agitation are limited. Quetiapine is a second-generation antipsychotic with a low affinity for dopamine receptors and a high affinity for serotonin receptors. It has been found to be associated with a shorter duration of delirium, reduced agitation, and higher rates of discharge to home after hospitalization [105].

Although antipsychotics may help manage symptoms of delirium or agitation, meta-analyses do not demonstrate any benefit in terms of outcomes such as symptom duration, severity, length of hospital stay, placement location, or mortality [106].

Benzodiazepines might play a limited role in the treatment of delirium. Since benzodiazepines have been found to increase the risk of developing delirium, their use should be avoided, except for selected indications such as alcohol withdrawal or when antipsychotic drugs are contraindicated. Benzodiazepines potentiate GABAa receptor function, a ligand-gated chloride channel, by increasing channel opening frequency and resulting in hyperpolarizations of neurons [107]. Among them, midazolam can be an effective treatment to control acute agitation, and it has been extensively studied in the ED setting. ED providers use IM midazolam for acute agitation due to its rapid onset of effects. An average onset of sedation has been showing at 15 min in patients receiving midazolam [108]. Limitation is the short duration of action ranging from 1 to 2 h. Lorazepam is commonly used in ED due to its rapid action and short half-life. In a double-blinded randomized trial conducted in ED, midazolam IV (median dose 5 mg) has been found to be as effective as droperidol (median dose 10 mg) in achieving sedation in ten minutes [109]. According to a trial performed in urban ED, the onset of sedation with midazolam is quicker compared to lorazepam or haloperidol, though the overall effectiveness of the three drugs seems to be comparable [110]. IM midazolam has been found to achieve more sedation effect in agitated ED patients at 15 min compared to olanzapine, ziprasidone, and haloperidol [111].

When feasible, the use of medications with anticholinergic properties should be avoided, or the dosage should be reduced. Alternatively, a medication with less anticholinergic strength could be used as a substitute, avoiding, for example, a tertiary amine such as amitriptyline [53]. Diphenhydramine should not be used for the treatment of the elderly. Its anticholinergic adverse effects can lead to a worsening of delirium and prolonged sedation [112].

Since antipsychotics and benzodiazepines may result in inadequate sedation, ketamine has emerged as an alternative solution in ED settings [113]. Ketamine is a dissociative anesthetic with a rapid onset of action (2–3 min), and it was used to provide analgesia for a wide variety of painful conditions [114]. However, there is limited information about its safety in older patients with delirium [40,115]. Dexmedetomidine is a sedative agent used in the ICU that showed a lower risk of causing delirium compared to midazolam but with no evidence in elderly ED patients.

The prescription of high-risk medications that can contribute to cognitive impairment described by the Beers criteria is common in ED. For this reason, a routine medication review is needed in the ED. Further research is needed on the safety of sedative drugs in geriatric patients, such as ketamine or dexmedetomidine. A nonpharmacological approach remains crucial to preventing delirium development. Pitkala et al. [116] demonstrated an improved health quality of life and faster alleviation of delirium by associating nonpharmacological strategy and avoidance of antipsychotics.

## 4. Limitations and Strengths

Since we conducted a narrative review, we provided an overview and synthesis of the existing literature topics on delirium in older ED patients. Given the heterogeneity of previous studies, we were not able to give a precise insight into the epidemiology of delirium in the ED. Secondly, most of the findings from previous reviews do not refer specifically to ED populations, making it difficult to apply them to the ED setting. Since no pharmacological treatment has been found to be effective in delirium without increasing the risk of prolonging delirium itself, we described the latest medication proposals without reporting a clear indication in the ED. We reviewed the most used delirium scales by comparing them for a better understanding of which might be the best for a busy environment.

## 5. Conclusions

Delirium is an often underrecognized public health issue, and it has a huge impact on older people’s health. It can affect older patients in the ED, leading to various adverse outcomes, including decreased long-term function, increased mortality, lengthened hospital stays, and increased medical costs. Patient management is challenging, especially when delirium is accompanied by agitation, which can lead to self-harm with necessary medical procedures. Several general risk factors contribute to delirium occurrence. Among them, advanced age, pre-existing cognitive impairment, underlying medical conditions, polypharmacy, sensory disruption, malnutrition, and infections are the most studied. A great number of ED assessment studies were conducted, but they are sparse and present limitations because of the variability of the patient population and the limit of the ED high-pressure environment. The 4T seems to be the best instrument for ED delirium assessment due to its quick administration and no need for specialized training or additional tests. Treatment of patients with delirium is complicated by the critical nature of their illness. Therefore, we conducted an updated revision, including the most recent literature, to analyze risk factors linked to the onset of delirium in ED patients and the management and treatment of ED elderly patients. A growing number of studies have been conducted on the prevention and management of delirium in ED settings, but little evidence is available to clinicians on the proper management of delirium, especially in the geriatric ED population.

## Data Availability

Not applicable.
